# Enterovirus A71 DNA-Launched Infectious Clone as a Robust Reverse Genetic Tool

**DOI:** 10.1371/journal.pone.0162771

**Published:** 2016-09-12

**Authors:** Chee Wah Tan, Han Kang Tee, Michelle Hui Pheng Lee, I-Ching Sam, Yoke Fun Chan

**Affiliations:** Department of Medical Microbiology, Faculty of Medicine, University of Malaya, 50603 Kuala Lumpur, Malaysia; The Scripps Research Institute, UNITED STATES

## Abstract

Enterovirus A71 (EV-A71) causes major outbreaks of hand, foot and mouth disease, and is occasionally associated with neurological complications and death in children. Reverse genetics is widely used in the field of virology for functional study of viral genes. For EV-A71, such tools are limited to clones that are transcriptionally controlled by T7/SP6 bacteriophage promoter. This is often time-consuming and expensive. Here, we describe the development of infectious plasmid DNA-based EV-A71 clones, for which EV-A71 genome expression is under transcriptional control by the CMV-intermediate early promoter and SV40 transcriptional-termination signal. Transfection of this EV-A71 infectious DNA produces good virus yield similar to *in vitro-*transcribed EV-A71 infectious RNA, 6.4 and 5.8 log_10_PFU/ml, respectively. Infectious plasmid with enhanced green fluorescence protein and Nano luciferase reporter genes also produced good virus titers, with 4.3 and 5.0 log_10_ PFU/ml, respectively. Another infectious plasmid with both CMV and T7 promoters was also developed for easy manipulation of *in vitro* transcription or direct plasmid transfection. Transfection with either dual-promoter infectious plasmid DNA or infectious RNA derived from this dual-promoter clone produced infectious viral particles. Incorporation of hepatitis delta virus ribozyme, which yields precise 3’ ends of the DNA-launched EV-A71 genomic transcripts, increased infectious viral production. In contrast, the incorporation of hammerhead ribozyme in the DNA-launched EV-A71 resulted in lower virus yield, but improved the virus titers for T7 promoter-derived infectious RNA. This study describes rapid and robust reverse genetic tools for EV-A71.

## Introduction

Enterovirus A71 (EV-A71) is the main causative agent of hand, foot and mouth disease (HFMD) in children. The clinical symptoms of HFMD are fever, rash on hands and feet, and oral ulcers [1]. Unlike other enteroviruses that cause HFMD, EV-A71 infections are also associated with severe neurological complications such as aseptic meningitis, brainstem encephalitis and acute flaccid paralysis [1–3]. Therefore, along with poliovirus, EV-A71 is a neurotropic enterovirus of great public health concern. To date, no vaccine or antiviral is available to prevent or treat EV-A71 infection [4].

EV-A71 consists of an approximately 7.4 kb positive strand RNA genome in an icosahedral capsid. The RNA genome consists of a single open reading frame, flanked by 5’ and 3’ untranslated regions with a poly(A) tail. The open reading frame encodes a polypeptide that is self-cleaved into structural proteins (VP1-VP4) and non-structural proteins (2A-2C and 3A-3D) [5]. A number of cellular receptors have been reported, including scavenger receptor class B2 [6], P-selectin glyprotein ligand-1 [7], heparan sulfate [8], annexin [9], vimentin [10] and sialic acid [11].

To study the gain and loss of function of viral genes, robust reverse genetic tools that allow genome-wide manipulation are needed. Such a genetic tool was first reported by Racaniello and Baltimore in 1981, in which the poliovirus genome was cloned into a pBR322 vector [12]. Since then, infectious cDNA clones for other viruses, such as dengue virus [13], coxsackieviruses [14], hepatitis A virus [15], polioviruses [16] and EV-A71 [17,18] have been reported. Traditionally, infectious cDNA clones for picornaviruses are cloned down-stream of a bacteriophage promoter, either SP6 or T7. This requires *in vitro* RNA synthesis and transfection or electroporation of infectious RNA into cells to obtain infectious viral particles. Many EV-A71 T7/SP6 promoter-driven infectious cDNA clones have been reported [6,17–21]. Preparation of T7/SP7 promoter-driven infectious clones is usually time-consuming and expensive. In this study, we have established a DNA-launched EV-A71 infectious clone which requires no *in vitro* RNA synthesis. This was achieved by cloning of the EV-A71 genome downstream of a RNA polymerase II promoter, cytomegalovirus (CMV) promoter, in a mammalian expression vector. We have successfully constructed multiple DNA-launched infectious clones equipped with unique self-cleavage ribozyme sequences to ensure precise 5’ and 3’ ends, and with multiple reporter genes. We also report a dual-promoter DNA-launched infectious clone with both eukaryotic and bacteriophage promoters. Our plasmid-based clones produced good virus titers similar to the traditional T7 promoter-driven infectious clones, over a shorter period of time. These DNA-launched EV-A71 infectious clones are robust research tools for study of EV-A71 pathogenesis, and can also be used as the basis for candidate DNA-based vaccines.

## Materials and Methods

### Cells, virus and plasmid

Rhabdomyosarcoma (RD, ATCC no.: CCL-136) cells and African green monkey kidney (Vero, ATCC no.: CCL-81) cells were obtained from American Type Culture Collection (ATCC, USA) and grown in Dulbecco’s Modified Eagle’s Medium (DMEM), supplemented with 10% fetal bovine serum (FBS). EV-A71 strain 41 (5865/SIN/000009, GenBank accession no. AF316321) was propagated in RD cells. Viruses were harvested when cells showed 70% cytopathic effect, freeze-thawed twice, and the virus-containing supernatant was kept at -80°C. The plasmid pCMV-TALER35 with the chikungunya virus genome was provided by Professor Andres Merits, University of Tartu, Estonia [22]. pCR-TOPO-XL cloning vector was purchased from Invitrogen, USA.

### Construction of EV-A71 T7 promoter-driven infectious cDNA clone

EV-A71 genomic RNA was extracted using QIAamp Viral RNA mini kit (QIAGEN, Germany) according to the manufacturer’s instructions. EV-A71 cDNA was synthesized using pEV71-R (S1 Table) using Superscript III reverse transcriptase (Invitrogen, USA). PCR was performed using pEV71-F and pEV71-R (S1 Table) using Q5 High-Fidelity DNA polymerase (NEB, USA). The resulting 7.4 kbp product was cloned into pCR-XL-TOPO vector and transformed into XL10-GOLD ultracompetent cells (Agilent Technologies, USA). The internal T7 promoter of pCR XL TOPO was removed using deletion PCR followed by T4 DNA ligase-T4 polynucleotide kinase-*Dpn*I (NEB, USA) treatment prior to transformation into XL10-GOLD ultracompetent cells (Agilent Technologies, USA). The resulting pT7-EV71 infectious clone was subjected to full-genome sequencing.

### Construction of EV-A71 CMV promoter-driven infectious cDNA clone

The EV-A71 genomic sequence was amplified by PCR using pEV71-F1, pEV71-R1, pEV71-F2 and pEV71-R2 (S1 Table) using Q5 High-Fidelity DNA polymerase (NEB, USA). pCMV-TALER35 was amplified using pCMV-F and pCMV-R (S1 Table). Gel-purified products were fused together with the Gibson assembly method (NEB, USA), according to the manufacturer’s instructions. A hepatitis delta virus (HDV) self-cleavage ribozyme sequence was inserted in between the EV-A71 poly(A)_25_ tail and simian virus 40 (SV40) termination signal by two rounds of anchor PCR using primers pHDV-F1, pHDV-R1, pHDV-F2 and pHDV-R2 (S1 Table). A hammerhead (HH) self-cleavage ribozyme sequence was inserted in between the CMV promoter and EV-A71 5’UTR by anchored PCR using primers pHH-F1, pHH-R1, pHH-F2 and pHH-R2 (S1 Table). The purified PCR products were then treated with T4 DNA ligase-T4 polynucleotide kinase-*Dpn*I (NEB, USA) treatment prior to transformation into XL10-GOLD ultracompetent cells (Agilent Technologies, USA). The resulting pCMV-EV71, pCMV-HH-EV71, pCMV-EV71-HDV and pCMV-HH-EV71-HDV infectious clones were subjected to full-genome sequencing.

Infectious plasmid DNA with both CMV and T7 promoters was constructed by anchored-PCR. PCR was performed using pCMV-T7-F and pCMV-T7-R primers with the pCMV-HH-EV71-HDV backbone. The PCR product was then treated with T4 ligase-T4 polynucleotide kinase-*Dpn*I before transformation into XL10-GOLD ultracompetent cells. The resulting pCMV-T7-HH-EV71-HDV infectious clone was subjected to DNA sequencing.

Enhanced green fluorescence protein (EGFP) and Nano luciferase (Nluc) genes were inserted in between 5’UTR and VP4 as previously described [17,23]. The EGFP sequence was amplified from pEGFP-N1 (Clonetech, USA) using pEGFP-F and pEGFP-R primers with Q5 High-Fidelity DNA polymerase (NEB, USA). The Nluc gene was amplified using pNluc-F and pNluc-R primers. The EV-A71 backbone was amplified using pEV71-EGFP-F and pEV71-EGFP-R primers. Two fragments were then digested with both *Age*I and *Hind*III, cloned, and transformed into XL10-GOLD ultracompetent cells (Agilent Technologies, USA). The resulting clones were subjected to DNA sequencing.

### *In vitro* RNA synthesis and transfection

Recombinant plasmids pT7-EV71 and pCMV-T7-HH-EV71-HDV were linearized using *Age*I and *Not*I (NEB, USA) respectively, followed by phenol:chloroform:isoamyl alcohol (25:24:1) and chloroform:isoamyl alcohol (24:1) purification. An aliquot of 5 μg of linearized DNA was used for *in vitro* RNA synthesis using the RiboMAX T7 large scale RNA synthesis kit (Promega, USA) according to manufacturer’s instructions. The *in vitro*-transcribed RNA was then purified using Illustra Microspin G-25 columns (GE Healthcare, UK). An aliquot of 2 μg of purified RNA was transfected into 5 × 10^5^ RD cells in a 6-well plate using TransIT-mRNA (MirusBio, USA) according to the manufacturer’s instructions. The inoculum was removed 4 hours post-transfection, and immediately replaced with fresh 10% FBS DMEM.

The DNA-launched infectious clone was purified using PureLink HiPure plasmid mini kit (Invitrogen, USA). For transfection of the DNA-launched infectious clone, 2 μg of plasmid DNA was transfected into 5 × 10^5^ RD cells in a 6-well plate using Lipofectamine LTX with PLUS Reagent (Invitrogen, USA) according to the manufacturer’s instructions. The inoculum was removed 4 hours post-transfection, and immediately replaced with fresh 10% FBS DMEM. EV-A71 infectious virus particles were harvested 72 hours post-transfection for subsequent analysis.

### Plaque assay

The plaque assay was carried out as previously described with minor modifications [21,24]. In brief, 10-fold serial diluted EV-71was inoculated into a 6-well plate pre-seeded with 5 × 10^5^ cells/well. The inoculum was removed after an hour post-infection, and replaced with DMEM supplemented with 0.8% carboxylmethylcellulose (Sigma, USA) and 2% FBS. The cells were fixed with 3.7% formaldehyde and stained with 0.5% crystal violet at 72 hours post-infection.

### Immunofluorescence assay

The EV-71 infected RD cells were loaded onto a poly-L-lysine coated glass slide and air-dried. The cells were then fixed with 3.7% formaldehyde for 10 min and permeabilized with 0.25% Triton-X-100 for 5 min. The cells were subsequently blocked with Image-iT FX signal enhancer (Invitrogen, USA) for 1 hour. EV-71 viral particles were immunostained with mouse anti-EV-71 monoclonal antibody (3324, Millipore, USA) as primary antibody and 1:200-diluted Alexa Fluor 488-labeled anti-mouse IgG (Invitrogen, USA) as the secondary antibody for an hour at 37°C. For nuclear visualization, cells were treated with 0.01% 4’,6-diamidino-2-phenylindole (DAPI, Sigma) for 7 min at room temperature. Immunofluorescence was detected with a fluorescence microscope.

### Luciferase assay

Luciferase assay was performed using Biolux Luciferase Assay kit (NEB, USA) according to manufacturer’s instructions. In brief, 20 μl of infected cell supernatant was loaded into CellCarrier-96 optic black plate (Perkin-Elmer, USA) and assayed with 50 μl of luciferase substrates. The luciferase activity was then measured using the GloMAX Multi Detection System (Promega, USA). The data presented as log_10_ relative light unit (RLU).

### Statistical analysis

The data presented are the means obtained from at least two independent biological replicates. Error bars represent the standard deviations of the means. Statistical significance was calculated using the independent *t*-test. A *P* value of <0.05 was considered statistically significant. All graphs and statistical tests were performed with GraphPad Prism 5 software (GraphPad Software, USA).

## Results

### Construction of recombinant CMV promoter-driven EV-A71 infectious clones

**Fig 1:**
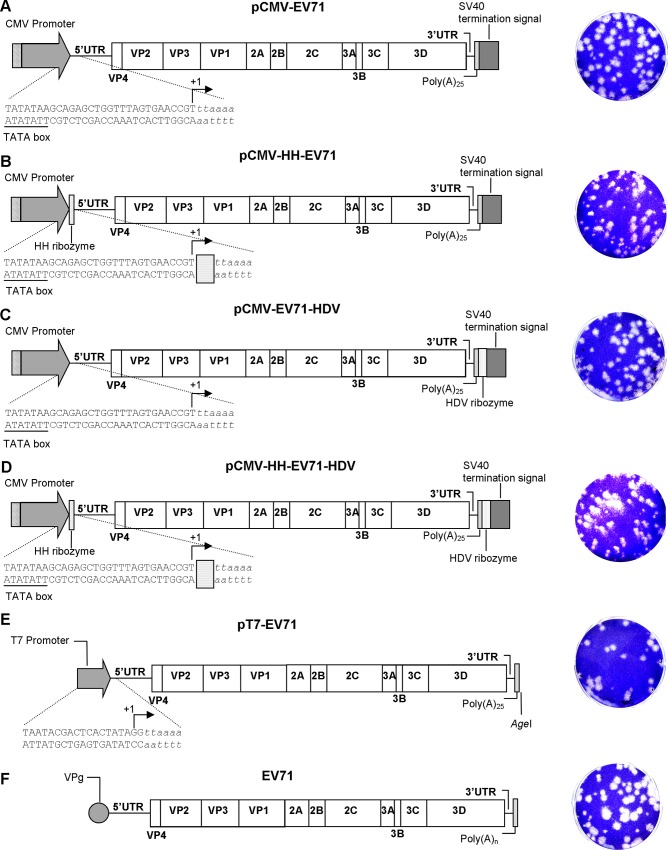
Schematic illustrations of CMV promoter-driven and T7 promoter-driven EV A71 infectious cDNA clones. The schematic illustrations of (A) pCMV-EV71, the CMV promoter-driven EV-A71 infectious clone; (B) pCMV-HH-EV71, the CMV promoter-driven EV-A71 infectious clone with the HH ribozyme before the 5’UTR; (C) pCMV-EV71-HDV, the CMV promoter-driven EV-A71 infectious clone with the HDV ribozyme after the EV-A71 poly(A)_25_ tail; (D) pCMV-HH-EV71-HDV, the CMV promoter-driven EV-A71 infectious clone with the HH ribozyme upstream of EV-A71 5’UTR and HDV ribozyme downstream of EV-A71 poly(A)_25_; (E) pT7-EV71, the T7 promoter-driven EV-A71 infectious clone; and (F) wild-type EV-A71. Italicized nucleotides indicate EV-A71 genomic DNA. Arrows indicate transcription start sites. The plaque morphologies of each clone-derived EV-A71 are shown in the right panel.

Based on the knowledge previously gained from constructing a T7/SP6-driven EV-A71 infectious clone [21], we sought to establish a convenient DNA-launched infectious cDNA clone driven by the CMV promoter for transient expression of EV-A71 genomic RNA. The EV-A71 genome (~7.4 kbp) was assembled downstream of a CMV promoter and upstream of a SV40 poly-adenylation termination signal using the Gibson assembly method. The successful construct has the EV-A71 5’UTR located immediately downstream of the CMV promoter and the SV40 poly-adenylation signal located downstream of the EV-A71 poly(A)_25_ tail (Fig 1A).

Addition or deletion of nucleotides at the 5’ and 3’ ends of clone-derived infectious transcripts may have deleterious effects on replication and are repaired at low frequency *in vivo* [16,25]. HH and HDV ribozymes were used to remove the excessive non-viral nucleotides from the EV-A71 transcripts from the 5’ and 3’ ends, respectively. Schematic illustrations of HH and HDV ribozymes are shown in S1 Fig. We successfully constructed DNA-launched EV-A71 infectious clones with either HH ribozyme inserted immediately upstream of the EV-A71 5’UTR or HDV ribozyme downstream of EV-A71 poly(A)_25_ tail, or both (Fig 1B–1D). A conventional T7 promoter-driven EV-A71 infectious cDNA in which the EV-A71 genome was cloned downstream of a T7 promoter was used for comparison in this study (Fig 1E). This T7 promoter-derived transcript carried two non-viral guanine residues at the 5’ end. All clones were subjected to full-genome sequencing and showed no additional mutations compared to the wild type virus.

### Characterization of the CMV promoter-driven EV-A71 infectious clones

All clones produced similar plaque morphology with 1.8–2.2 mm mean plaque size (Fig 1 and Table 1). Wild type EV-A71 produced multiple plaque morphology with mean plaque size of 2.6 mm ± 1.1, suggesting a mixed virus population. In fluorescence microscopy analysis, all clone-derived EV-A71 yielded positive signal using EV-A71-specific monoclonal antibody. All the DNA-launched infectious clones yielded infectious EV-A71 (Fig 1A–1E), with virus titers of 5 to 7 log_10_ PFU/ml at 3 days post-transfection (Table 1). Transfection of pCMV-EV71 plasmid DNA yielded comparable virus titers to the T7-derived infectious RNA, at 6.4 and 5.8 log_10_ PFU/ml, respectively. The presence of the HDV ribozyme after the EV-A71 poly(A)_25_ tail significantly increased the virus titers to 7.3 log_10_ PFU/ml, when compared to the pCMV-EV71. However, the presence of the HH ribozyme upstream of the EV-A71 5’UTR of the DNA-launched infectious clones reduced the overall virus production. These data suggest that greater precision at the 3’ end improved the infectivity of DNA-launched infectious RNA.

**Table 1:** Characterization of EV-A71 infectious clones by virus titers and plaque size.

EV-A71 clones	P_0_ viral titer (log_10_ PFU/ml)*	Plaque size (mm)*
pT7-EV71	5.80 ± 0.16	1.8 ± 0.5
pCMV-EV71	6.44 ± 0.07	2.2 ± 0.8
pCMV-HH-EV71	5.30 ± 0.25 (*P* < 0.05)^a^	2.1 ± 0.6
pCMV-EV71-HDV	7.29 ± 0.27 (*P* < 0.05)^a^	2.2 ± 0.6
pCMV-HH-EV71-HDV	5.06 ± 0.27 (*P* < 0.05)^a^	2.0 ± 0.4
WT	N.A	2.6 ± 1.1

* The data are presented as means ± standard deviation of at least two biological replicates.

^a^ Statistically significant compared with pCMV-EV71.

N.A indicates not applicable.

### Construction and characterization of the CMV promoter-driven EV-A71 reporter viruses

**Fig 2:**
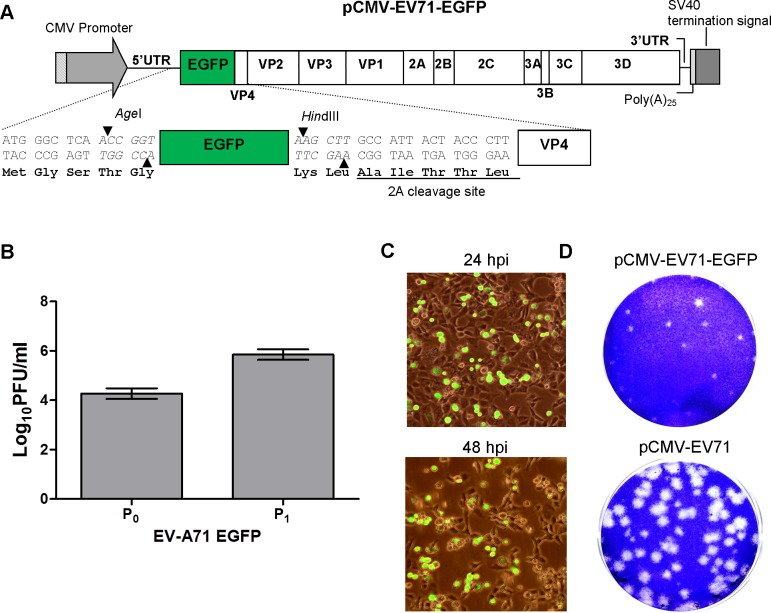
Schematic illustration and characterization of pCMV-EV71-EGFP. (A) EGFP was cloned downstream of the EV-A71 5’UTR and upstream of the EV-A71 VP4 gene. EGFP was inserted through *Age*I and *Hin*dIII restriction enzyme sites. A 2A cleavage site was inserted after the EGFP gene. (B) The virus titers in log_10_ PFU/ml of pCMV-EV71-EGFP at P_0_ and P_1_. (C) RD cells were infected with EV-A71-EGFP at an MOI of 0.1, and green fluorescence was captured 24 and 48 hours post-infection. (D) Plaque morphologies of clone-derived EV-A71 and EV-A71-EGFP are shown.

**Fig 3:**
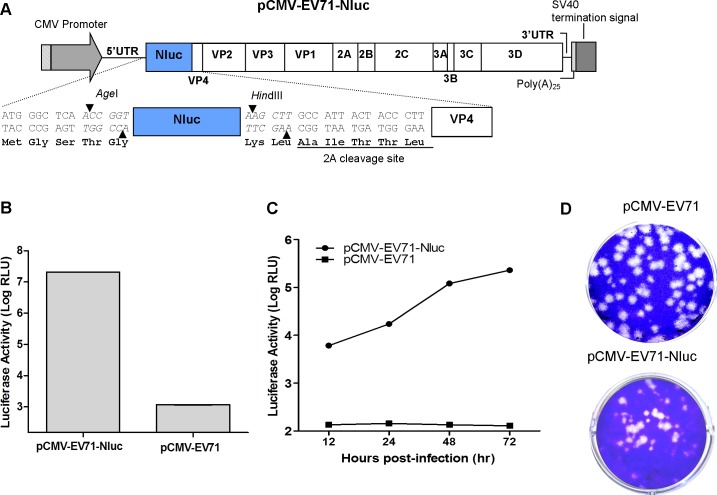
Schematic illustration and characterization of pCMV-EV71-Nluc. (A) Nluc was cloned downstream of the EV-A71 5’UTR and upstream of the EV-A71 VP4 gene. Nluc was inserted through *Age*I and *Hin*dIII restriction enzyme sites. A 2A cleavage site was inserted after the Nluc gene. (B) RD cells were infected with EV-A71-Nluc at an MOI of 1, and the luciferase activity was determined upon 48 hours post-infection. (C) RD cells were infected with EV-A71-Nluc at an MOI of 0.1, and the luciferase activity was determined at 12, 24, 48, and 72 hours post-infection. (D) Plaque morphologies of clone-derived EV-A71 and EV-A71-Nluc are shown.

A virus carrying a reporter will enable tracking and monitoring of virus during infection. The cDNA clone of the EV-A71 reporter virus was constructed by inserting the reporter gene between the EV-A71 5’UTR and the N-terminus of VP4, flanked by two unique restriction digestion sites, *Age*I and *Hind*III. These two unique restriction sites were first introduced into pCMV-EV71 before construction of the reporter clone. In order to enable cleavage of the reporter gene from VP4, an EV-A71 2A protease cleavage site (AITTL) was introduced between the reporter gene and the VP4 gene. The EGFP (714bp, encoding 238 amino acids) or Nluc (513bp, encoding 171 amino acids) gene was engineered into the pCMV-EV71 infectious clone at the *Age*I and *Hind*III sites to produce pCMV-EV71-EGFP (Fig 2A) and pCMV-EV71-Nluc (Fig 3A) reporter virus infectious clones.

To test whether the resulting reporter infectious clones are replication-competent, pCMV-EV71-EGFP or pCMV-EV71-Nluc plasmid DNA was transfected into RD cells using Lipofectamine LTX for 72 hours. Re-infection of the RD cells with EV-A71-EGFP yielded up to 5.7 log_10_ PFU/ml (Fig 2B) and bright green fluorescence signals were detected by fluorescence microscopy (Fig 2C). For EV-A71-Nluc, the luciferase activity detected was 7.3 log_10_ RLU upon 48 hours post-infection at an MOI 1(Fig 3B). The luciferase activity of EV-A71-Nluc (MOI = 0.1) was detected at 12 hours post-infection and increased to 5.4 log_10_ RLU at 72 hours post-infection (Fig 3C). Both reporter viruses yield smaller plaques compared to EV-A71 without reporters (Figs 2D and 3D).

### Construction and characterization of a dual-promoter EV-A71 infectious cDNA clone

**Fig 4:**
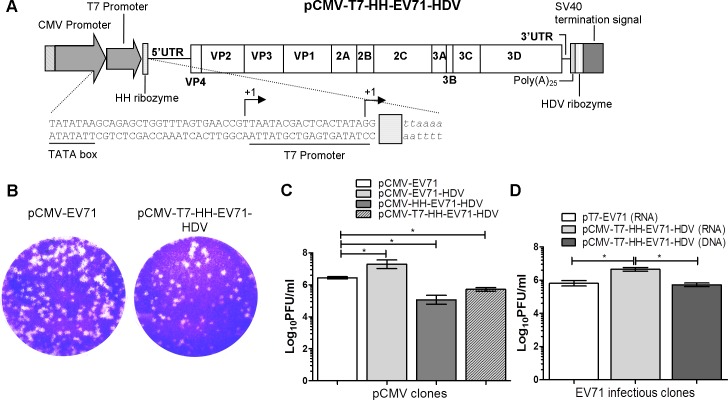
Schematic illustration and characterization of pCMV-T7-HH-EV71-HDV dual-promoter EV-A71 infectious clone. (A) To construct the dual-promoter infectious clone, a T7 promoter was inserted right after the transcription start site of the CMV promoter and upstream of the HH ribozyme sequence. Arrows indicate transcription start sites for CMV and T7 promoters. CMV promoter-derived transcripts carry the T7 promoter sequence and will be removed by HH ribozyme to ensure a precise 5’ end. T7 promoter-derived transcripts carry the HH ribozyme upstream of the EV-A71 5’ UTR to ensure removal of non-viral nucleotides. (B) The plaque morphologies of the pCMV-EV71 and pCMV-T7-HH-EV71-HDV at 72 hours post-infection. The virus titers at 72 hours post-transfection of the (C) DNA and (D) *in vitro* synthesized RNA into RD cells using Lipofectamine LTX and TransIT-mRNA, respectively. Error bars indicate standard deviations of the mean values.

To avoid the need for multiple infectious clones, we constructed an infectious clone with both mammalian expression CMV promoter and a bacteriophage T7 promoter. Infectious viral particles can be produced by transfection of plasmid DNA or *in vitro* T7 polymerase-transcribed infectious RNA into cells. The T7 promoter sequence was introduced into pCMV-HH-EV71-HDV between the CMV transcription start site and the HH self-cleavage ribozyme sequence (Fig 4A). To test whether the dual-promoter EV-A71 cDNA clone is replication-competent, equal amounts of pCMV-EV71, pCMV-EV71-HDV, pCMV-HH-EV71-HDV and pCMV-T7-HH-EV71-HDV plasmid DNA were transfected into RD cells as described previously. Virus from pCMV-T7-HH-EV71-HDV showed similar plaque morphology to pCMV-EV71 (Fig 4B). As shown in Fig 4C, pCMV-T7-HH-EV71-HDV yielded virus titers of 5.7 log_10_ PFU/ml while pCMV-HH-EV71-HDV yielded 5.1 log_10_ PFU/ml.

To examine the infectivity of T7 promoter-derived EV-A71 transcripts in this dual-promoter construct, infectious RNA was synthesized from pT7-EV71 and pCMV-T7-HH-EV71-HDV using T7 polymerase, and transfected into RD cells for 72 hours. The presence of HH ribozyme upstream of the EV-A71 5’ UTR will remove the two non-viral guanine residues at the 5’ end of the T7 promoter-derived transcripts. The transcripts with HH ribozyme produced higher virus titers of 6.7 log_10_ PFU/ml compared to the transcripts without HH ribozyme derived from pT7-EV71, which produced only 5.8 log_10_ PFU/ml (Fig 4D). The results here showed that the same vector can be used for plasmid DNA transfection or *in vitro* transcription.

### *In vitro* replication kinetics of clone-derived EV-A71

**Fig 5:**
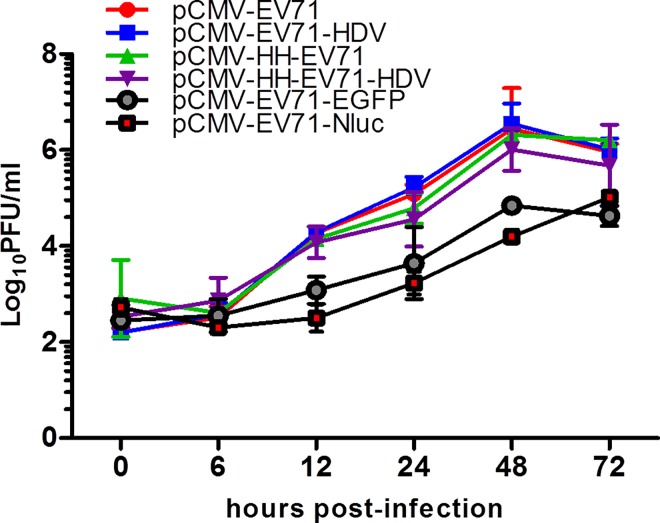
Replication kinetics of clone-derived EV-A71 in RD cells. RD cells were infected with various clone-derived EV-A71 at an MOI of 0.1. The viral titers in log_10_ PFU/ml were determined at 0, 6, 12, 24, 48 and 72 hours post-infection using plaque assay. Error bars indicate standard deviations around the means.

To determine the replication kinetics of all the clone-derived EV-A71, RD cells were infected with various clone-derived EV-A71 at an MOI of 0.1 and the viruses were harvested at 0, 6, 12, 24, 48 and 72 hours post-infection for viral titration. As shown in Fig 5, all clone-derived EV-A71 exhibited similar replication kinetics and achieved highest viral titers of up to 6 log_10_ PFU/ml 48 hours post-infection, with the exception of EV-A71 with reporter genes. Both EV-A71 reporter viruses exhibited lower replication compared to EV-A71 without reporter genes. EV-A71- EGFP showed similar replication compared to EV-A71-Nluc, with peak viral titers of about 4.8 log_10_ PFU/ml and 5.0 log_10_ PFU/ml at 48 and 72 hours post-infection, respectively.

## Discussion

Reverse genetic tools enable the study of loss and gain of function of viral genes to understand viral pathogenesis, and aid in the development of vaccines and therapeutics. Since the first poliovirus infectious clone was developed [12,26], multiple infectious clones for many viral pathogens have been developed, including EV-A71 [17,18,23]. DNA-launched infectious clones have been reported for a number of viruses, including dengue virus [27,28], Japanese encephalitis virus [28,29] and chikungunya virus [22]. DNA-based infectious viral vectors may also be used as novel vaccine candidates [30–32]. For poliovirus, such clones are available by cloning of the poliovirus cDNA under transcriptional control of SV40-late promoter and SV40 termination signal [33,34]. The foot-and-mouth disease virus (FMDV) infectious plasmid DNA was transcriptionally controlled by both RNA polymerase I and II promoters which enabled production of high amounts of positive-stranded RNA [35]. An EV-A71 reverse genetic system under RNA polymerase I promoter was also reported previously [36].

**Fig 6:**
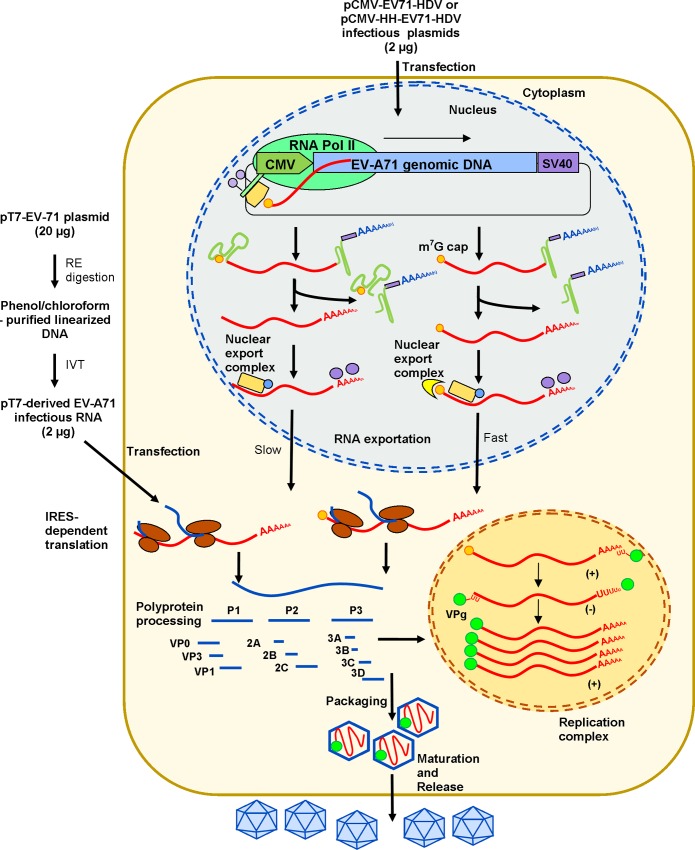
Schematic illustration of the mechanism of EV-A71 DNA-launched infectious clone. The viral RNA requires exportation to the cytosol for cap-independent translation by mRNA export complexes. The HH ribozyme removes the m^7^G cap and thus ensures precise 5’ ends of the EV-A71 transcripts. The HDV ribozyme removes the SV40 termination signal and ensures precise 3’ ends of viral RNA. Viral RNA is exported into the cytosol by RNA export complexes. The m^7^G cap is required for nuclear RNA export. Removal of the cap diminishes the RNA exportation efficiency and therefore, reduces overall virus production. *In vitro*-transcribed infectious RNA is transfected into the cytosol for IRES-dependent translation. All newly synthesized positive-strand viral RNA are VPg-linked. RE indicates restriction enzyme; and IVT indicates *in vitro* transcription.

In this study, we have developed multiple DNA-based EV-A71 expression vectors placed under transcriptional control of the CMV immediate-early promoter and SV40 transcription termination signal, allowing EV-A71 genomic RNA to be transcribed by cellular RNA polymerase II. The likely mechanism of a DNA-launched infectious clone is highlighted in Fig 6. RNA polymerase II-derived EV-A71 genomic RNA are 7-methylguanylate (m^7^G)-capped [37]. Transcription of the CMV promoter yields m^7^G-capped EV-A71 genomic RNA with a non-viral uracil residue at the 5’ end within the nucleus of the cell. The presence of the m^7^G cap did not significantly alter both EV-A71 infectivity and *in vitro* translation using HeLa lysates (S2 Fig). This may suggest that the m^7^G cap at the 5’ end of EV-A71 RNA transcript has no role in viral translation [38], and will be removed during EV-A71 negative strand synthesis in the cytosol. Moreover in picornaviruses, viral RNA is translated into viral proteins by cap-independent translation [39]. The m^7^G-capped viral RNA will be exported out to the cytosol by multiple export factors and complexes for IRES-dependent translation [40–43]. Heterogeneous nuclear ribonucleoprotein (hnRNP) complexes are one of the factors involved in mRNA nuclear export [42] while hnRNP A1 was reported to be important for EV-A71 cap-independent translation [44]. All newly synthesized positive strand genomic RNA produced during viral replication are VPg-linked.

The presence of non-viral nucleotides at the 5’ and 3’ ends of enterovirus genomic RNA could reduce viral infectivity [16,25,45,46]. The HH ribozyme is essential for precise 5’ end generation [16,35]. The HH ribozyme belongs to a family of small endonucleolytic ribozymes that catalyze the cleavage of its own phosphodiester backbone by means of a trans-esterification reaction [47,48]. The HDV ribozyme has been used to accurately generate 3’ ends of transcribed viral RNA [49–52]. HDV is a RNA satellite virus (1.7 kb RNA genome) of hepatitis B virus and has genomic and anti-genomic versions of a small ribozyme. These ribozymes have been used in multiple DNA-based viral expression vectors [16,49–52]. Our data demonstrated that the presence of a self-cleavage HH ribozyme sequence upstream of the EV-A71 5’UTR significantly reduced the virus yield in the DNA-launched infectious clones. The presence of the HH ribozyme removes the 5’ m^7^G cap viral RNA in the nucleus, and therefore could reduce the rate of RNA exportation into cytosol for translation [40]. This data suggests that the 5’ m^7^G cap is important for DNA-launched infectious clones. *In vitro* synthesized infectious RNA derived from the T7 promoter of the dual-promoter clone was equipped with self-cleavage HH ribozyme at the 5’ end. Transfection of this infectious RNA with precise 5’ ends to the cytosol of RD cells increased viral infectivity. The non-viral nucleotides are removed at low frequency by VPg-pUpU priming during positive-strand RNA synthesis [16]. Hence, the presence of HH ribozyme will improve cleavage of non-viral nucleotides resulting in enhanced infectivity of RNA-launched infectious clones.

Multiple studies suggest that transfection of *in vitro* synthesized infectious RNA with precise 3’ ends showed no difference in virus titers when compared with infectious RNA with additional non-viral nucleotides at the 3’ end [45,53]. However, our data demonstrated that the presence of a self-cleavage HDV ribozyme sequence at the 3’ end significantly increased overall virus yield of the DNA-launched infectious clone after transfection. Without the HDV ribozyme, the pCMV-EV71-derived infectious RNA carried 122 non-viral nucleotides from the SV40 termination signal and this resulted in dramatic reduction of the viral infectivity.

We have also described the construction of DNA-launched EV-A71 infectious clones with EGFP and Nluc reporter genes cloned in between 5’UTR and VP4 gene, as previously reported [17,23]. A 2A protease cleavage sequence was introduced to release the reporter protein from the EV-A71 polyprotein. Replication of the reporter viruses was slower compared with viruses without reporter genes. The presence of foreign genes in the picornavirus RNA genome can significantly affect viral RNA packaging in the rigid viral capsid [54,55].

The presence of the potential splice sites in the viral genomic RNA could possibly make it difficult for the DNA-launched infectious clone to express in the nucleus [56], which may explain why these clones are not widely available. The DNA-based EV-A71 infectious clones produced infectious EV-A71 viral particles and exhibited similar replication rates to wild-type EV-A71, implying that no splicing occurred during post-transcriptional modification. This data is consistent with the intron prediction analysis using GENSCAN that no intron exists in the EV-A71 genomic RNA (data not shown).

In this study, we constructed multiple robust EV-A71 infectious plasmid-based clones under transcriptional control of RNA polymerase II. The plasmid-based clones produced comparable virus titers to the traditional T7 promoter-driven infectious clones. Any observable difference in virus yields between both infectious clones could be a result of different transfection efficiency due to use of different cell lines and reagents [57–59]. We also performed transfection of DNA-launched infectious clone pCMV-EV71 and T7 promoter-driven infectious RNA in other cell lines such as Vero and U87MG cells, and found variable transfection efficiency in these cells as expected. The DNA-launched infectious clone was less effective in Vero cells, but showed similarly good efficiency in RD and U87MG cells (S3 Fig). It also takes 2 days to construct our DNA-launched clones, compared to the T7 promoter-driven infectious clones that require up to 4 days. Importantly, this infectious DNA technology could allow rapid and more robust study of EV-A71 pathogenesis. This infectious clone can also be used as a candidate DNA-based vaccine that stably expresses live attenuated EV-A71.

## Supporting Information

S1 Fig The EV-A71-specific *cis*-acting ribozymes. Predicted secondary structure of the *cis*-acting (A) HH and (B) HDV ribozymes attached to the 5’ and 3’ ends of the EV-A71 genome, respectively. The EV-A71 genome is shown in italics. Arrows indicate ribozyme cleavage sites.
(TIF)

S2 Fig The effects of m^7^G cap on EV-A71 protein synthesis and infectivity. m^7^G-capped and uncapped EV-A71 infectious RNA was synthesized using mMESSENGER mMACHINE kit (Ambion, USA) and RiboMAX large scale RNA synthesis system (Promega, USA), respectively. (A) The capped and uncapped viral RNAs were transfected into Vero cells using TransIT-mRNA (MirusBio, USA). The viral titers were quantitated 4 days post-transfection by plaque assay. The data are presented in log_10_ PFU/ml. Error bars indicate standard deviations around the means. (B) *In vitro* translation was performed using 1-step human coupled IVT kit (Pierce, USA) with 1 μg of capped and uncapped RNA at 30°C for 4 hours. The viral protein expression was determined by western blot analysis using EV-A71-specific monoclonal antibody.
(TIF)

S3 Fig The efficacy of plasmid-based and RNA-launched infectious clones in RD, Vero and U87-MG cells. An aliquot of 2 μg of pCMV-EV71 and T7 promoter-derived RNA were transfected into (A) RD, (B) Vero and (C) U87-MG cells for 4 hours. The media were replaced with fresh 10% FBS DMEM or EMEM, followed by 72 hours incubation. The viruses were harvested for subsequent plaque assay.
(TIF)

S1 Table Primers used in preparation of EV-A71 infectious clones. (DOCX)
