# Supplementary figures and images for: Enterovirus A71 DNA-Launched Infectious Clone as a Robust Reverse Genetic Tool
Source: PLoS One. 2016 Sep 12;11(9):e0162771. doi: 10.1371/journal.pone.0162771 (PMC5019408; doi:10.1371/journal.pone.0162771)

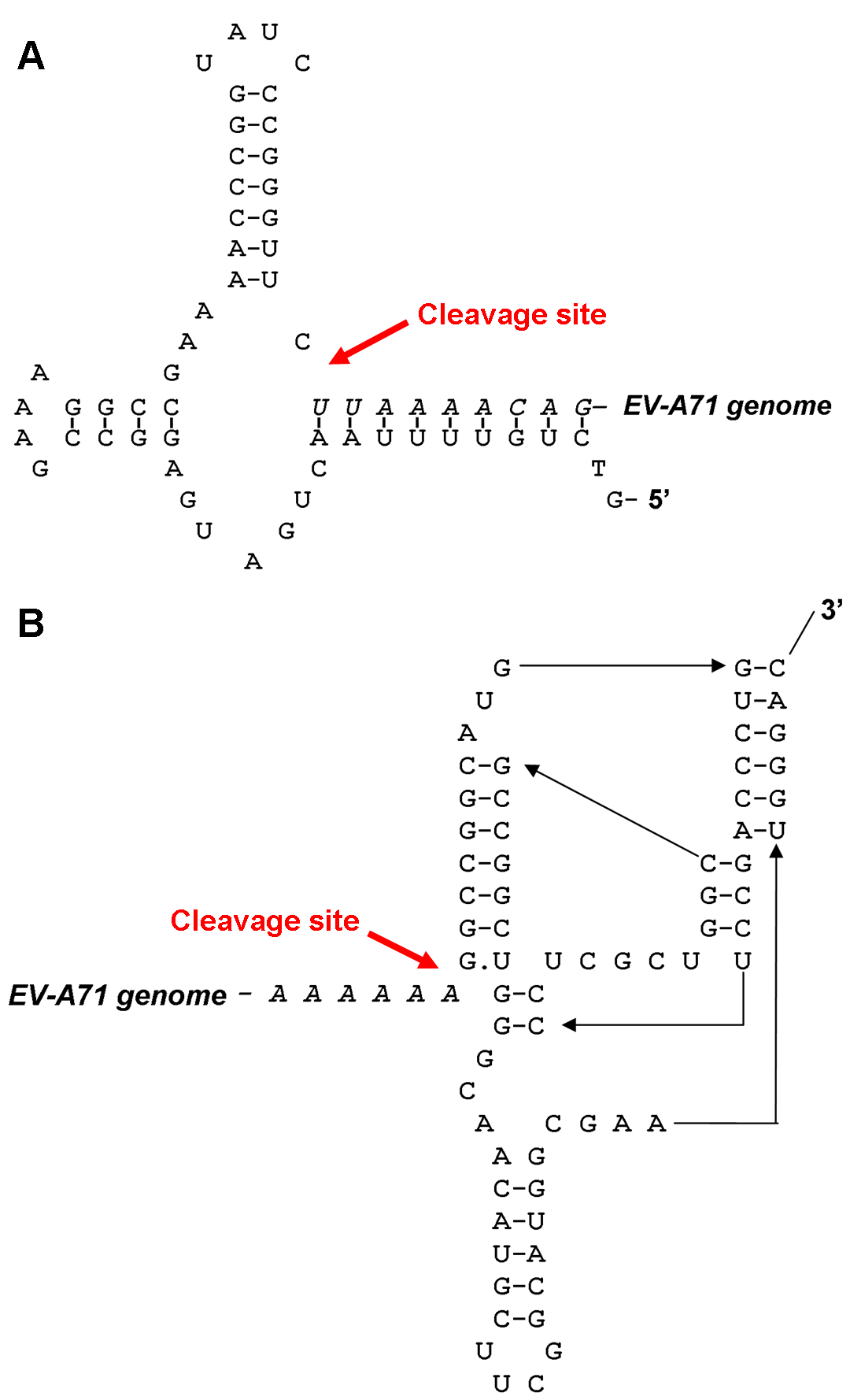

Supplement: S1 Fig — Predicted secondary structure of the cis-acting (A) HH and (B) HDV ribozymes attached to the 5’ and 3’ ends of the EV-A71 genome, respectively. The EV-A71 genome is shown in italics. Arrows indicate ribozyme cleavage sites. (TIF) [file pone.0162771.s001.tif]

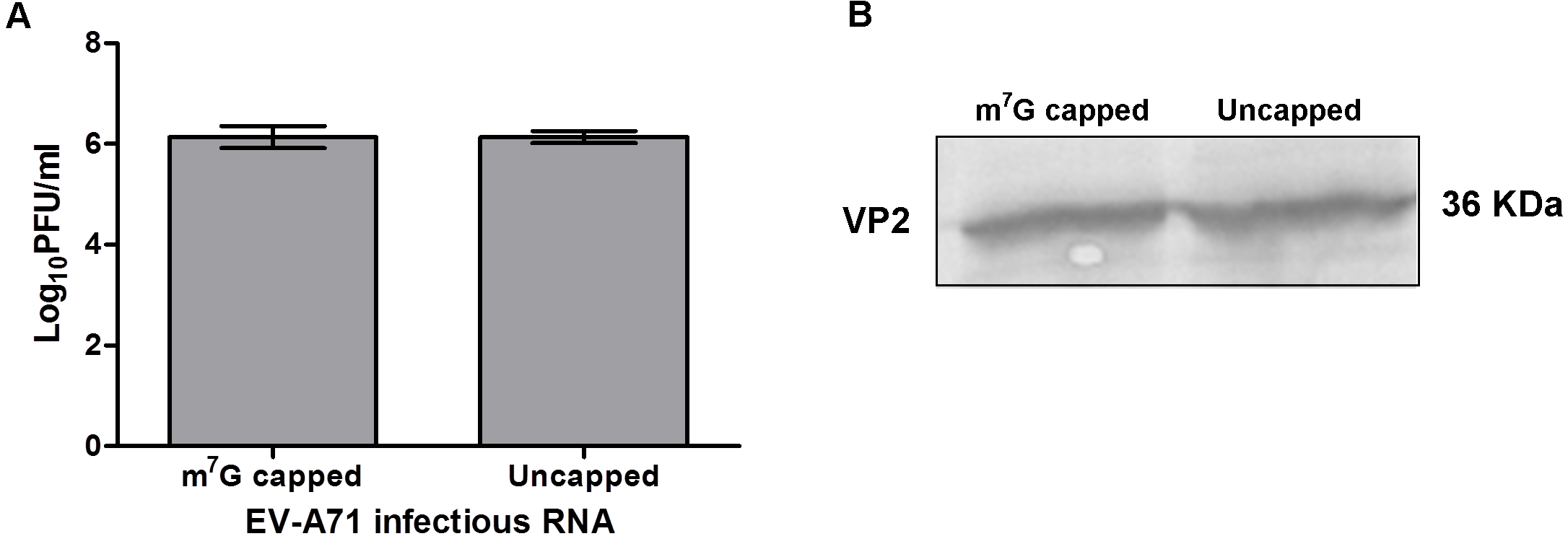

Supplement: S2 Fig — m7G-capped and uncapped EV-A71 infectious RNA was synthesized using mMESSENGER mMACHINE kit (Ambion, USA) and RiboMAX large scale RNA synthesis system (Promega, USA), respectively. (A) The capped and uncapped viral RNAs were transfected into Vero cells using TransIT-mRNA (MirusBio, USA). The viral titers were quantitated 4 days post-transfection by plaque assay. The data are presented in log10 PFU/ml. Error bars indicate standard deviations around the means. (B) In vitro translation was performed using 1-step human coupled IVT kit (Pierce, USA) with 1 μg of capped and uncapped RNA at 30°C for 4 hours. The viral protein expression was determined by western blot analysis using EV-A71-specific monoclonal antibody. (TIF) [file pone.0162771.s002.tif]

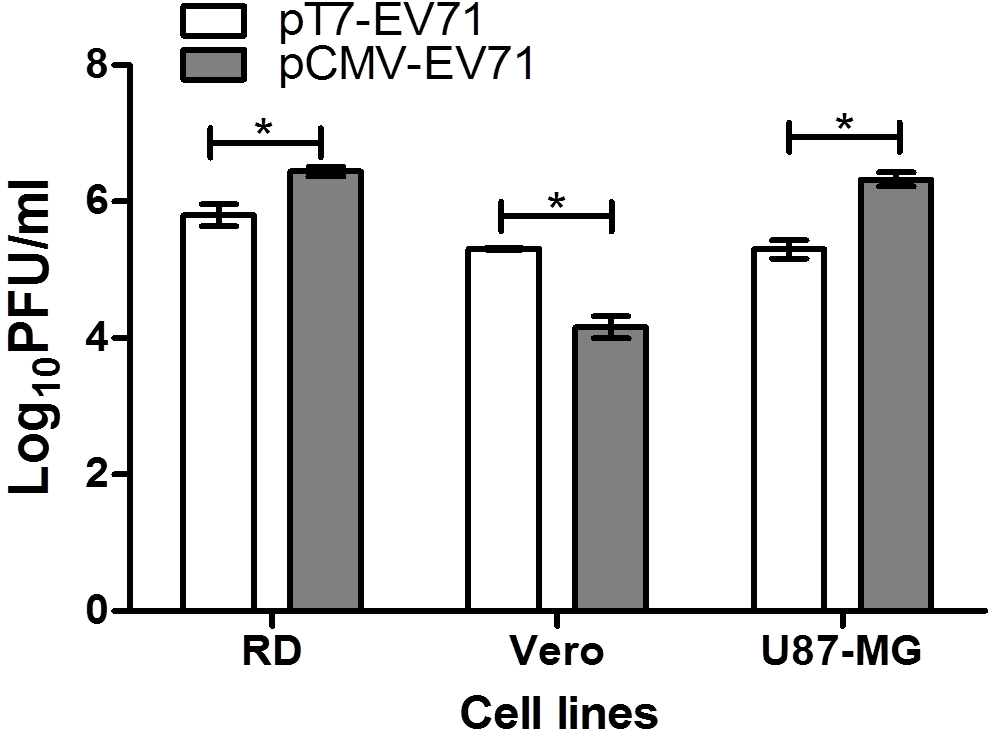

Supplement: S3 Fig — An aliquot of 2 μg of pCMV-EV71 and T7 promoter-derived RNA were transfected into (A) RD, (B) Vero and (C) U87-MG cells for 4 hours. The media were replaced with fresh 10% FBS DMEM or EMEM, followed by 72 hours incubation. The viruses were harvested for subsequent plaque assay. (TIF) [file pone.0162771.s003.tif]
