# Supplementary material for: Enterovirus A71 DNA-Launched Infectious Clone as a Robust Reverse Genetic Tool
Source: PLoS One. 2016 Sep 12;11(9):e0162771. doi: 10.1371/journal.pone.0162771 (PMC5019408; doi:10.1371/journal.pone.0162771)
Supplement: S1 Table — (DOCX) [file pone.0162771.s004.docx]

**Supporting Table**

**S1 Table. Primers used in preparation of EV-A71 infectious clones**

| **Primers** | **Primer sequence (5’ 🡪 3’)** |
| --- | --- |
| pEV71-F | ACGCGTAACGTAATACGACTCACTATAGGTTAAAACAGCCTGTGGGTTGC |
| pEV71-R | ACCGGTTTTTTTTTTTTTTTTTTTTTTTTTTGCTATTCCGGTTATAAC |
| pCMV-F | AAAAAAAAAAAAAAAAAAAAAAAAAAAAAAGCGGCCGCGAATTGTTGTGTT |
| pCMV-R | TGCAACCCACAGCTGTTTTAAACGGTTCACTAAACCAGCTCTG |
| pEV71-F1 | AGAGCTGGTTTAGTGAACCGTTTAAAACAGCTGTGGGTTGCAC |
| pEV71-R1 | ACACTATACCAACTACACCATGTTGACACC |
| pEV71-F2 | GGGGCATCTTAAGGTGTCAACATGGTG |
| pEV71-R2 | AACAACAACAATTCGCGGCCGCTTTTTTTTTTTTTTTTTTTTTTTTTTTTTT |
| pHDV-F1 | CGGTAATGGCGAATGGGACGGCGGCCGCGAATTGTTGTTGTTAAC |
| pHDV-R1 | AGGCTGGGACCATGCCGGCCTTTTTTTTTTTTTTTTTTTTTTTTTTTTTT |
| pHDV-F2 | CAACATTCCGAGGGGACCGTCCCCTCGGTAATGGCGAATGGGACGGCGGC |
| pHDV-R2 | CCCAGCCGGCGCCAGCGAGGAGGCTGGGACCATGCCGGCCTTTTT |
| pHH-F1 | CCGAAAACCCGGTATCCCGGGTTCTTAAAACAGCCTGTGGGTTGCAC |
| pHH-R1 | CCTTTCGGCCTCATCAGAGAGACACGGTTCACTAAACCAGCTCTG |
| pHH-F2 | TTAACTGATGAGGCCGAAAGG |
| pHH-R2 | AACAACGGTTCACTAAACCAGCT |
| pEV71-EGFP-F | TACAAGCTTGCCATTACTACCCTTGGCTCACAGGTGTCTACTCAGCGATC |
| pEV71-EGFP-R | CACACCGGTTGAGCCCATGTTTGATTGTATTGAGGG |
| pEGFP-F | TCAACCGGTGTGAGCAAGGGCGAGGAGC |
| pEGFP-R | GGCAAGCTTGTACAGCTCGTCCATGCCGAG |
| pNluc-F | TCAACCGGTGTCTTCACACTCGAAGATTTCGTTGGGGAC |
| pNluc-R | GGCAAGCTTCGCCAGAATGCGTTCGCACAGCCGCCAGCC |
| pCMV-T7-F | CACTATAGGGTGTTTTAACTGATGAGGCCG |
| pCMV-T7-R | AGTCGTATTAACGGTTCACTAAACCAGCTC |
